# Supplementary material for: A meta-ethnographic systematic review of women’s experiences of homelessness in high income environments
Source: PLoS One. 2026 Jan 20;21(1):e0339371. doi: 10.1371/journal.pone.0339371 (PMC12818621; doi:10.1371/journal.pone.0339371)
Supplement: S1 Appendix — (PDF) [file pone.0339371.s001.pdf]

ETHOS Light

EUROPEAN TYPOLOGY OF HOMELESSNESS AND HOUSING EXCLUSION

A Harmonised Definition of Homelessness for Statistical Purposes

Sometimes referred to as ETHOS 'Light', this is a version of the ETHOS typology developed in the context of a 2007 European Commission study: *Measurement of Homelessness at European Union Level.* It is a harmonised definition of homelessness for statistical purposes. It is a pragmatic tool for the development of homelessness data collection, rather than a conceptual and operational definition to be used for a range of policy and practice purposes.

OPERATIONAL CATEGORY		LIVING SITUATION		DEFINITION
1	People living rough	1	Public spaces / external spaces	Living in the streets or public spaces without a shelter that can be defined as living quarters
2	People in emergency accommodation	2	Overnight shelters	People with no place of usual residence who move frequently between various types of accommodation
3	People living in accommodation for the homeless	3	Homeless hostels	Where the period of stay is time-limited and no long-term housing is provided
		4	Temporary accommodation	
		5	Transitional supported accommodation	
		6	Women's shelters or refuge accommodation	
4	People living in institutions	7	Health care institutions	Stay longer than needed due to lack of housing
		8	Penal institutions	No housing available prior to release
5	People living in non-conventional dwellings due to lack of housing	9	Mobile homes	Where the accommodation is used due to a lack of housing and is not the person's usual place of residence
		10	Non-conventional buildings	
		11	Temporary structures	
6	Homeless people living temporarily in conventional housing with family and friends (due to lack of housing)	12	Conventional housing, but not the person's usual place of residence	Where the accommodation is used due to a lack of housing and is not the person's usual place of residence
