# Supplementary material for: A meta-ethnographic systematic review of women’s experiences of homelessness in high income environments
Source: PLoS One. 2026 Jan 20;21(1):e0339371. doi: 10.1371/journal.pone.0339371 (PMC12818621; doi:10.1371/journal.pone.0339371)
Supplement: S2 Appendix — (DOCX) [file pone.0339371.s002.docx]

# ASSIA Search Strategy

#1 women

ABSTRACT,TITLE Women? OR girl? OR woman OR ladies OR lad* OR female? OR **mainsubject.exact.explode("Women and children")** OR **mainsubject.exact.explode("Women")** OR **mainsubject.exact.explode("Females")**

AND

2# Homeless

ABSTRACT,TITLE homeless? OR homeless* OR "no fixed address" OR "no fixed abode" OR "underhouse*" OR "roofless*" OR "seeking shelter" OR unhouse* OR "sleeping rough" OR "rough sleep*" OR "unstabl* hous*" OR "housing instability" OR "precarious* hous*" OR runaway? OR unsheltered OR "Street People" OR "Street person*" OR shelter* OR Indigent* OR Transient? OR "homeless persons" OR **mainsubject.exact.explode ("Homelessness") OR mainsubject.exact.explode ("Homeless people")**

3# experience

ABSTRACT, TITLE Experience? OR understanding? OR view? OR feelings OR Perspective? OR phenomen* OR explor* OR theme? OR thematic OR interview* OR 'Focus group?' OR 'action research' OR participatory OR ethnograph* OR narrative? OR ethnomethodolog* OR Interpretation? OR Comprehension

4# HIC

ABSTRACT,TITLE **mainsubject.exact.explode ("Western countries")** OR Antigua* OR "Antigua and Barbuda*" OR Aruba* OR Australia* OR Austria* OR Bahamas OR Bahrain* OR "British Virgin Islands" OR Brunei OR Darussalam OR Canada OR "Cayman Islands" OR "Channel Islands" OR Chile OR Croatia* OR Curacao OR Cyprus OR Czechia OR "Czech Republic" OR Denmark OR Estonia* OR "Faroe Islands*" OR Finn* OR France OR German* OR Gibralta* OR Greece OR "French Polynesia" OR Greenland* OR Guam OR "Hong Kong" OR Chin* OR Hungar* OR Iceland* OR Eire OR Ireland OR "ROI" OR "Isle of Man" OR Israel* OR Ital* OR Japan* OR "Republic of Korea" OR "South Korea*" OR Korean OR Kuwait* OR Latvia* OR Liechtenstein OR Lithuania* OR Luxembourg* OR Iceland* OR Macao* China OR Malta OR Monaco OR Nauru* OR Netherlands OR "New Caledonia" OR "New Zealand*" OR "Northern Mariana Islands" OR Norway OR "North America" OR Oman OR Panama OR Poland OR Portugal OR "Puerto Rico" OR Qatar OR Romania* OR "San Marino" OR "Saudi Arabia*" OR Seychelles OR Singapore* OR "Sint Maarten" OR Slovak* OR Slovenia* OR Spain OR "St Kitts and Nevis" OR "St Martin" OR Swed* Switzerland OR "Trinidad* and Tobago*" OR "Turks and Caicos Island*" OR Caribbean OR "USA" OR "United Arab Emirates" OR "United Kingdom" OR "UK" OR "United States" OR "The United States of America" OR Uruguay* OR "Virgin Island*" OR "England" OR "Scotland" OR "Wales"

DATABASE SPECIFIC MODIFICATION

LIMITS applied using ASSIA

**AND subt.exact(("female" OR "women") AND "homeless people")**

**as in women or homeless people thesarus terms ARE focus of article narrows results from 16953 results to 275**
