# Supplementary material for: A meta-ethnographic systematic review of women’s experiences of homelessness in high income environments
Source: PLoS One. 2026 Jan 20;21(1):e0339371. doi: 10.1371/journal.pone.0339371 (PMC12818621; doi:10.1371/journal.pone.0339371)
Supplement: S3 Appendix — (DOCX) [file pone.0339371.s003.docx]

| **Study ID** | **Study Title** | **Country** | **HIC** | **Aim of study** | **DESIGN** | **Study setting/context** | **Definition of homeless used in paper** | **Definition of homeless** | **Sample Size** | **Theoretical approach: ontology/epistemology/** |
| --- | --- | --- | --- | --- | --- | --- | --- | --- | --- | --- |
| Bazari 2018 | ‘The Thing that Really Gets Me Is the Future’: Symptomatology in Older Homeless Adults in the HOPE HOME Study | USA | Y | To characterize the experience, understanding, and management of physical, psychological, social(e.g., loneliness), and existential (e.g., regret, loss of dignity) symptoms among older homeless adults | Semi structured interviews | Unsheltered units | Y | HUD homeless definition 2009 | 20 participants | Positivist - rating scales involved in inclusion criteria and design. Interview topic guide iterative generation, analysis 'thematic' |
| Benbow 2019 | Spaces of Exclusion: Safety, Stigma, and Surveillance of Mothers Experiencing Homelessness | Canada | Y | Critical narrative study is to understand the experiences of social exclusion in the lives of mothers experiencing homelessness in Ontario | Interviews | Shelter and second-stage housing for pregnant and parenting young mothers, and the other was a shelter for women who were experiencing intimate partner violence. | N | not explicitly referenced | 26 mothers | Critical narrative analysis underpinned by intersectional/social justice/feminist ontology |
| Benyamin 2022 | Homeless in their own home’: the case of home-deprived single mothers | Israel | Y | Not explicitly stated as aim - foreground experience of israeli homeless women | interviews | Protest/various transient settings | Y | feminist/structural context: Passaro/Watson and Austerberry | 10 single mothers | Clearly feminist although npt Explicitly articulated |
| Biederman2013 | Homeless women’s experiences of social support from service providers | USA | Y | To examine homeless women’s interactions with service providers the degree to which these interactions are perceived as social support. | in-depth semistructured interviews | A winter emergency shelter and homeless drop in day center served as the study sites. | Y | US Department of Housing and Urban Development (HUD) definition at the time | 20 women | Phenomenological |
| Biederman 2016* Revisit of 2013 data set | The voice of patients and families Desired Destinations of Homeless Women: Realizing Aspirations Within the Context of Homelessness | USA | Y | To understand homeless women’s aspirations by posing the following research questions: 1. What are homeless women’s hopes for the future? 2. How do homeless women perceive their future? | secondary data analysis of qualitative interview data (Biederman 2013) with different foci | Winter emergency shelter and homeless drop in day center served as the study sites | N | not explicitly referenced | 20 women | Phenomenological |
| Bimpson 2022 | Governing homeless mothers: the unmaking of home and family | UK | Y | To understand the experiences of homeless women in relation and to the impact of homelessness on family life. | Qualitative interviews | Refuge and other trusted outreach organisations | N | not explicitly referenced: women at various stages of accessing provision based on UK legislation. Major subject of discussion of paper - but not an explicit inclusion/exclusion | 26 women | Not explicitly named but evidently based on references and framing intersectional and feminist perspective |
| Bowstead | Spaces of safety and more-than-safety in women’s refuges in England | UK | Y | Explores women’s refuges as spaces of safety, and of more-than safety, aim of facilitating ‘a deeper understanding of the complexities of the lives of research participants - women in refuges | Qualitative interviews | Refuges and refuge workers - multiple locations | N | not explicitly referenced | 20 women | Feminist participatory engagement |
| Borghi 2023 | The perceived impact of homelessness on health during pregnancy and the postpartum period: A qualitative study carried out in the metropolitan area of Nantes, France | France | Y | The objective of our study was to describe how the health of homeless women who are pregnant or in the postpartum period is impacted, according to them, by their physical, social, and healthcare surroundings. | Qualitative interviews, quantitative survey data: demographic and health statistics | Shelter, homeless drop in various clinic sites | Y | Ethos light | 26 women of which 20 homeless per ETHOS 6 are homeless adjacent - social colleague | Not explicity discussed beyond thematic analysis and semi structured interviews |
| Bretherton 2020 | Women’s Experiences of Homelessness: A Longitudinal Study | UK | Y | To add to the existing evidence on women’s experiences of homelessness and second to add to emergent debates on whether gender is associated with differentiated trajectories through homelessness - study was larger longnitudinal survey this is data from women in survey | longitudinal study six-monthly intervals, over a two-year period | Program evaluation in cities | N | not explicitly clear - references ETHOS and MPHASIS projects | 47 lone women | Not explicitly discussed |
| Cameron 2016 | From pillar to post: homeless women's experiences of social care. | UK | Y | To identify how their experiences and needs changed over this time. The aim was to gain a fuller understanding of their needs, including their social care needs, as a means to understand how best to support women to access, and maintain engagement with, support service | longitudinal case study- two years of data collection, 3 interviews recruitment | City | N | not explicitly discussed 'insecurely housed' | White British (27) with four women describing themselves as White European; two as Black African; and five as mixed race | Not explicitly discussed - positivist - rating scales involved in inclusion criteria and design. Interview topic guide iterative generation, analysis 'thematic' |
| Carey 2022 | “Hell on Earth”: Single mothers’ experiences of temporary accommodation in London and its impact on their mental health | UK | Y | What are single mothers’ experiences of living in temporary accommodation in London like and what impact does it have on their mental health? | Single interview with single mothers in temporary accommodation in east London, recruited via wide range of source | City | N | Not explicityly discussed | 12 single mothers |  |
| Cooper 2015 | Time Seizures and the Self: Institutional Temporalities and Self-preservation Among Homeless Women | USA | Y | Documents temporalities of homelessness as experienced by many homeless people today, those living in the midst of an urban ‘‘services ghetto’’—where social service organizations abound, but such organizations fail to coordinate the provision or timing of services, producing an incoherent multiplicity of offerings and schedules | 2 years of team ethnographic fieldwork | City | N | not explicitly discussed | 61 women | Not explicitly discussed |
| Debska and Mostowka 2021 | The stranded Cinderella and the wandering rascal. Two narratives of female housing exclusion | Poland | Y | To investigate how the intersections of homelessness, gender, motherhood, lack of family support etc. shape the participants’ lives, biographies,and self-images. | Qualitative interviewse | Rural and urban | Y | references annual count in Poland, references critique of this Homelessness or housing exclusion used interchangeably | 2 case studies - 2 women | Feminist |
| Fotheringham 2013 | ‘A place to rest’: the role of transitional housing in ending homelessness for women in Calgary, Canada | Canada | Y | To determine the role of transitional housing in ending homelessness for women and to explore how gender-specific experiences of homelessness may inform housing service delivery models. | Participatory study | City | Y | Canadian context: the specific housing situation and the duration/frequency of homelessness | 9 women | Not explicitly discussed |
| Gonyea 2017 | Older homeless women’s identity negotiation: agency, resistance, and the construction of a valued self | USA | Y | to address this gap through an in-depth investigation of the intersectionality of gender, age and homelessness for older women who find themselves without stable housing framework informed by intersectionality and symbolic interactionism, to understand how they talk about their identities and non-identities in an effort to negotiate and control their own sense of self. A central aim of the study was to gain a deeper understanding of how older homeless women engage in ‘identity work’ to address the tension between how others view them and how they view themselves and construct a sense of a valued life and self. | In-depth semi-structured interviews spaced apart by 2 months to 7 months | Non-profit older homeless organization | N | not explicitly = references HUD | 20 older women | Phenomenological/intersectional approach recognition of power structures |
| Gultekin 2014 | Voices From the Street: Exploring the Realities of Family Homelessness | USA | Y | The specific aims of this study were to conduct focus groups with mothers experiencing homelessness and their caseworkers, separately, to (a) explore individual pathways into homelessness, (b) understand the day-to-day experience of living in an emergency shelter and the process of rehousing, (c) identify real and perceived barriers for families attempting to reestablish stable housing, and (d) understand the impact of homelessness on families' overall health and well-being. | Qualitative; focus groups | Detroit, Michigan USA homeless services support setting | Y | HUD 2009 | 13 mothers 5 caseworkers | Feminist participatory action research |
| Kirkman2015 | ‘I just wanted somewhere safe’: Women who are homeless with their children | Australia | Y | What it meant to women to be homeless with their children | Qualitative | Supported accom as result of homelessness, not 'crisis' accommodation | Y | In this article, we adopt the cultural definition of homelessness (Chamberlain and MacKenzie, 1992) on which the ABS based its statistical definition which was applied | 11 mothers and 1 grandmother | qualitative |
| Lewinson2014 | Traumatic Transitions: Homeless Women’s Narratives of Abuse, Loss, and Fear | USA | Y | To explore types of trauma and adversity prior to and during housing at budget hotels. In this qualitative study, narrative and categorical content-analysis approaches were conducted to identify common themes across women’s narratives. | Qualitative semi structured interview | Budget hotels in Gwinnett County of Metropolitan | Y | U.S. Code defines persons as homeless if they lack a regular residence, if they sleep in a place not intended for human habitation, or if they stay in emergency or transitional shelter (42 USC § 11302). | 21 | Interrelated theoretical perspectives, specifically variations of trauma theory and feminist theory and empowerment perspectives that recognize the impact of oppression and the role of mutual relationships and self-determination, |
| Mayock2015 | It's just like we're going around in circles and going back to the same thing …’: The Dynamics of Women's Unresolved Homelessness | Ireland | Y | An in-depth account of the lives and experiences of homeless women in Ireland | Biographical interviews | Homeless hostels (both single and mixed gender), domestic violence refuges, long-term supported housing, transitional | N | not explicitly defined - focus on **long-term homelessness** | 26 women | Not explicitly discussed, hinted at feminist perspective and ethnographic.. |
|  |  |  |  |  |  | accommodation and other temporary living situations. |  |  |  |  |
| Menih 2020 | ‘COME NIGHT-TIME, IT’S A WAR ZONE’: WOMEN’S EXPERIENCES OF HOMELESSNESS, RISK AND PUBLIC SPACE | Australia | Y | Explores constructions of ‘risk’ in the lives of women experiencing homelessness in Brisbane, Australia. | Ethnographic data, including in-depth interviews, informal conversations and participant observation field notes over total period of 10 months over two phases. | Brisbane various sites | N | not explicit | 10 | feminist |
| McGrath2023 | Social capital and women’s narratives of homelessness and multiple exclusion in northern England | UK | Y | To examine how women with co-occurring homelessness, substance misuse and poor mental health utilise social capital and to what extent it is helpful or hindering to them | Qualitative interviews, case study analysis | Urban north of England recruited via homeless drop in | N | Homeless, rough sleeping or couch surfing, not explicit reference | 20 |  |
| Moore2014 | Coping with homelessness: an expectant mother’s homeless pathway | UK | Y | Aim of the study was to examine housing pathways rather than the experiences within one place | Phenomenological interviewing approach | Individual housing context | N | not explicit | 1 woman | Phenomenology |
| Phipps2020 | More than a house: Women’s recovery from homelessness in Australia | Australia | Y | This study investigated women's experiences of exiting homelessness and examined the factors that influenced recovery | Qualitative interpretive study and photo elicitation | City | Y | In this study, women's homelessness was defined Australian Bureau of Statistics, 2014) | 10 | Phenomenology |
| Price and Glorney2022 | The challenge to survive: trauma, violence and identity in the lived experience of homeless women | UK | Y | To understand how females who had recently been street homeless made sense of their lived experience, | Semi structured interviews. Phenological analysis | Three homelessness projects in a provincial city | Y | lack of secure housing - time since and ' street homelessness' | 7 | IPA |
| Salem and MaPham | Understanding Health Needs and Perspectives of Middle-Aged and Older Women Experiencing Homelessness | USA | Y | The study was to assess perspectives among prefrail and frail, middle-aged and older homeless women. | Mixed methods Focus groups of specific subsets of women and qualitative interviews | This study was conducted in a large, urban drop-in day center | Y | An individual was defined (United States Department of Housing and Urban Development, 1995). | 20 women | Not explicitly discussed |
| Salem2013 | At a Crossroads: Reentry Challenges and Healthcare Needs among Homeless Female Ex-Offenders | USA | Y | To understand the unique gendered experiences of homeless female ex-offenders, in the context of healthcare needs, types of health services sought, and gaps | Descriptive, qualitative study conducted with focus group methodology | Residential drug treatment -ex-offenders enrolled in a residential drug treatment program | N | not explicitly defined | 16 women who were parolees/ probation | Pedagogy, politics and interpretive inquiry to cross-pollinate. The collaborative approach of community-based participatory methods (CBPR) |
|  |  |  |  | in order to help them achieve a smooth transition post prison release |  |  |  |  |  |  |
| Salsi2017 | Occupational needs and priorities of experiencing homelessness | Canada | Y | To gain knowledge on the occupational lives of women experiencing homelessness | A participatory, descriptive mixed-methods study design was used | Homeless shelter - some STA and some LTA | Y | Canadian Observatory on Homelessness (2012) | 19 women | Community-based participatory approach |
| Schmidt2015 | Trajectories of women’s homelessness in Canada’s 3 northern territories | Canada | Y | The study was designed to learn about the barriers and supports experienced by homeless women in the North when accessing mental health care, shelter, housing and other services; and to inform the work of northern service providers and policy advocates in a position to implement adjustments in their praxis. | 2-year, multilevel action research project using semi structured interviews | 3 sites in Canadian Northern Territories | N | not explicitly discussed | 41 women | Not explicity discussed - action research |
| Sutherland2022 | Older women’s perceptions of the impact of homelessness on their health needs and their ability to access healthcare | Australia | Y | This study explored the healthcare needs and barriers to health services in older homeless women in the Perth metropolitan area, Western Australia. | Mixed methods - Twenty-two older women experiencing homelessness completed a questionnaire and semi-structured interview | Seven homeless support services | Y | Institute of Health and Welfare. Homelessness and Homelessness Services [Internet]. Canberra (AUST): AIHW; 2020 [cited 2021 Jan 24]. | 22 women | not explicitly discussed |
| Tutty2014 | I Built My House of Hope: Abused Women and Pathways Into Homelessness | Canada | Y | The current study focused on women’s narratives of their journeys into homelessness. | Semi- structured interviews | VAW centres | Y | Tutty Defining Homelessness Novac, 2006; Tutty, Ogden, & Weaver-Dunlop, 2007). Homelessness varies on a continuum from narrow to broad (Begin, Casavant, & Miller Chenier, 1999). |  | Not explicitly discussed in depth, mentions intersectionality and feminism |
| Van Berkum | Where to now?’ Understanding the landscape of health and social services for homeless women in London, Ontario, Canada | Canada | Y | To understand a network of health and social services accessed by women experiencing homelessness, and how individuals navigate these services. | Participatory PhotoVoice methodology | Drop-in centre supporting women experiencing homelessness in a midsized urban centre | N | not explicity discussed | 6 women | This project is guided by critical feminist intersectional theory. Critical Social Theory |
| Warburton2018 | Homelessness Pathways for Australian Single Mothers and Their Children: An Exploratory Study | Australia | Y | To explore homeless Australian mothers’ pathways into and out of homelessness, their specific needs and the services and supports | Semi-structured interviews | To strengthen the findings, collateral data were collected Metropolitan welfare agencies | N | not explicitly defined | 15 mothers | Not explicitly discussed |
| Wilson2015 | House to House, Shelter to Shelter: Experiences of Black Women Seeking Housing After Leaving Abusive Relationships | USA | Y | This pilot study explores the narratives of Black mothers currently residing at an emergency intimate partner violence shelter to discover their experiences in seeking housing after leaving abusive relationships with a focus on housing instability andmental health. | Qualitative descriptive narrative research design | Emergency IPV shelter | N | not explcitly discussed | 5 women | Not explicitly discussed - constructivist |
