# Supplementary material for: A meta-ethnographic systematic review of women’s experiences of homelessness in high income environments
Source: PLoS One. 2026 Jan 20;21(1):e0339371. doi: 10.1371/journal.pone.0339371 (PMC12818621; doi:10.1371/journal.pone.0339371)
Supplement: S4 Appendix — (DOCX) [file pone.0339371.s004.docx]

| Study ID | Was there a clear  statement of the aims of  the research? | Is a qualitative  methodology  appropriate? | Was the research  design appropriate to  address the aims of the  research? | Was the recruitment  strategy appropriate to  the aims of the  research? | Was the data collected in  a way that addressed the  research issue? | Has the relationship  between researcher and  participants been  adequately considered? | Have ethical issues been  taken into consideration? | Was the data analysis  sufficiently rigorous? | Is there a clear statement  of findings? | How valuable is the  research? |
| --- | --- | --- | --- | --- | --- | --- | --- | --- | --- | --- |
| Bazari 2018 | yes | yes | yes | yes | yes | dont know | yes | dont know | yes | only study of its kind in review (or literature I know of), |
| Benbow 2019 | yes | YES | YES | YES | YES | YES | YES | YES | YES | valuable implications for policy and practice for nurses |
| Benyamin 2022 | dont know | YES | YES | YES | YES | NO | NO | Dont know | YES | Valuable highly located |
| Biederman 2013 | yes | YES | YES | YES | YES | NO | YES | dont know | yes | Valuable highly located |
| Biederman 2016*same study dataset as 2013 | yes | YES | YES | YES | YES | NO | YES |  |  |  |
| Bimpson 2022 | yes | YES | YES | YES | YES | NO | YES | dont know | yes | Valuable highly located |
| Borghi | yes | YES | Dont know | Dont know | YES | NO | YES | Dont know | YES | Valuable highly located |
| Bowstead | yes | YES | YES | YES | YES | Dont know | YES | YES | YES | Valuable highly located |
| Bretherton 2020 | yes | YES | YES | YES | YES | Dont know |  | YES | YES | Valuable highly located |
| Cameron 2016 | yes | YES | YES | YES | YES | Dont know |  | dont know | yes | Valuable highly located |
| Carey 2022 | yes | YES | YES | YES | YES | YES | YES | YES | YES | Valuable highly located |
| Cooper 2015 | yes | yes | yes | Dont know | yes | yes | YES | Dont know | NO | this is valuable but it’s a highly 'located' piece |
| Debska and Mostowka 2021 |  | Dont know | Dont know | Dont know | YES | Dont know | YES | Dont know | NO | authors interpretation of archetypes. Valuable  exposition of context  No ethical approval board noted - only ethics from funder. |
| Fotheringham2013 | yes | YES | YES | YES | YES | YES | YES | Dont know | YES | This is a photovoice study and not solely qualitative interviews. |
| Gonyea2017 | yes | YES | YES | YES | YES | Dont know | YES | dont know | YES | Valuable highly located |
| Gultekin2014 | yes | YES | YES | YES | YES | YES | YES | dont know | YES | Valuable highly located |
| Kirkman2015 | yes | YES | YES | YES | YES | Dont know | YES | dont know | YES | children route in, 50$ incentive |
| Lewinson2014 | yes | YES | YES | YES | YES | Dont know | YES | dont know | YES | Valuable highly located data |
| Mayock2015 | yes | YES | YES | YES | YES | YES | YES | dont know | YES | Valuable highly located data |
| McGrath 2023 | yes | YES | YES | YES | YES | Dont know | YES | dont know | YES | Valuable highly located data |
| Menih 2021 | yes | YES | YES | YES | YES | YES | YES | dont know | YES | Enormously valuable detailed piece |
| Moore2014 | yes | YES | YES | Dont know | YES | Dont know | YES | dont know | YES | single individual IPA  valuable but highly contextual |
| Phipps 2021 A | yes | YES | YES | YES | YES | YES | YES | dont know | YES | Valuable highly located data |
| Phipps2021 B same dataset as A | yes | YES | YES | YES | YES | YES | YES | dont know | YES |  |
| Price and Glorney2022 | yes | YES | YES | YES | YES | Dont know | YES | dont know | YES | Valuable highly located data |
| Salem and MaPham | yes | YES | YES | YES | YES | Dont know | YES | dont know | YES | focus groups included only those frail/prefrail -= valuable for broader health interest ? But skeweed re experiences of homelessness |
| Salem2013 | yes | YES | YES | YES | YES | Dont know | YES | dont know | YES | small highly located sample |
| Salsi2017 | yes | YES | YES | YES | YES | Dont know | YES | dont know | yes | Valuable highly located data |
| Schmidt2015 | yes | YES | YES | YES | YES | Dont know | YES | dont know | yes | Valuable highly located data |
| Sutherland2022 | yes | YES | YES | YES | YES | Dont know | YES | dont know | yes | Valuable highly located data |
| Tutty2014 | yes | YES | YES | YES | YES | Dont know | YES | dont know | yes | Enormously valuable detailed piece |
| Van Berkum | yes | YES | YES | YES | YES | Dont know | YES | dont know | yes | this is a photovoice study and not solely qualitative interviews. |
| Warburton2018 | yes | YES | YES | YES | YES | Dont know | YES | dont know | yes | Valuable highly located data |
| Wilson2015 | yes | YES | YES | YES | YES | Dont know |  | dont know | yes | Valuable highly located data |
