# Supplementary material for: A meta-ethnographic systematic review of women’s experiences of homelessness in high income environments
Source: PLoS One. 2026 Jan 20;21(1):e0339371. doi: 10.1371/journal.pone.0339371 (PMC12818621; doi:10.1371/journal.pone.0339371)
Supplement: S5 Appendix — (DOCX) [file pone.0339371.s005.docx]

| **Author Year** | **2nd order= Paper Authors Metaphors/Concepts/Themes** | **underpinning line of argument (brief)** |
| --- | --- | --- |
| Bazari 2018 | Existential, psychological, and social symptoms caused as much distress as physical symptoms 2. Childhood abuse leads to enduring symptomatology in older adulthood | #Precarity #Surviving Violence and Exclusion #Existing with Risk |
| Benbow 2019 | overarching finding of **unsafe space**s represents the unique forms of exclusion from safety participants experienced in public and private spaces. Emerging out of this category are two intertwined subcategories of (a) **exclusion from safety and (b) stigma: public surveillance and discrimination.** | #Precarity #Surviving Violence and Exclusion #Existing with Risk # Parenting whilst homeless |
| Benyamin 2022 | #1Economic dimensions: ‘There Is Food We Eat. There Is No Food; We Don’t Eat’ #2 2. Emotional dimensions: ‘Alone Against Everything #3 3. Jenny: ‘I Feel Like I am Living in Boxes’ #4. Sarit: ‘I Was a Homeless in My Own House’ #5Rachel: ‘I Felt I Had Nothing to Lose. Either I Become a Squatter or I Wind Up on The Streets #6. Organized Resistance: ‘There will Not Be Abandoned Buildings and Children Without Shelter 7# | #Precarity #Surviving Violence and Exclusion #Existing with Risk # Parenting whilst homeless |
| Biederman 2016 | #1 connectedness through therapist relationship #2connectedness through homelesspeers #3 women described their potential to arrive at the destination of ***significant*** while homeless | #Precarity #Surviving Violence and Exclusion #Existing with Risk |
| Biederman 2013 | Experiences of social support or lack of | #Precarity #Surviving Violence and Exclusion #Existing with Risk # Fracturing Identity |
| Bimpson 2022 | #1 Housing and children’s social care: conflicting welfare policies #2 Safeguarding children; blaming mothers 3#Governing homeless mothers: undermining home-making | #Precarity #Surviving Violence and Exclusion #Existing with Risk # Parenting whilst homeless # Fracturing Identity |
| Borghi 2023 | Theme 1: The overall perceived negative impact of homelessness on all aspects of health (mental health, physical health, and social well-being)  Theme 2: The positive influence of social and professional support on health during pregnancy and the postpartum periodTheme 3: Having access to suitable housing, being independent, and being in good health | #Precarity #Surviving Violence and Exclusion #Existing with Risk # Parenting whilst homeless |
| Bowstead 2015 | Womens experiences of safety, and of more-than safety, complexity | #Precarity #Surviving Violence and Exclusion #Existing with Risk |
| Bretherton 2020 | Four pathways through homelessness identified for women in study #1 life as usual: this group tended to self-exit from homelessness,  #2 Long periods on the margins sustained and recurrent homelessness  #3Negative pathways due to health One, even where access to treatment was being facilitated, movement away from homelessness had not occurred. #4 constant negative churn group of nine women, characterised by recurrent and sustained homelessness and higher support needs, whose situation remained a constant. | #Precarity #Surviving Violence and Exclusion #Existing with Risk # Fracturing Identity |
| Cameron 2016 | #1# Relationships with support workers #2 having a worker of a similar age who had been through comparable experiences and could therefore empathise #3 Fragmentation- service disconnects #4 conditionality of supports | #Precarity #Surviving Violence and Exclusion #Existing with Risk |
| Carey 2022 | 4 themes Neglect and abuse within a powerful, unjust system; Futility, entrapment and cycles of suffering; Mothering against the odds; and Surviving and resisting in the face of adversity. | #Precarity #Surviving Violence and Exclusion #Existing with Risk # Parenting whilst homeless |
| Cooper 2015 | #1 shaping of subjectivity and self by homelessness  # 2dignity and respect socially situated aspects of self that homeless people struggle to maintain and negotiate, often at great cost #3 Focusing on ‘‘getting by’’ rather than on ‘‘getting out’’ | #Precarity #Surviving Violence and Exclusion #Existing with Risk # Fracturing Identity |
| Debska and Mostowka 2021 | Complex constructed narratives of two archetypes derived from research | #Precarity #Surviving Violence and Exclusion #Existing with Risk |
| Fotheringham 2013 | Gender-specific experiences of homelessness #1 Trauma. 2# Don’t fit gender role expectations - stereotypes and stigma =low self-esteem, #3 women were in agreement that they could not have gone from homelessness directly into permanent housing; the third and final theme. | #Precarity #Surviving Violence and Exclusion #Existing with Risk |
| Gonyea 2017 | 1)individuals may become engulfed by a homeless self-. 2) important was embracing identities that connected them to the social world and gave them a sense of valued self. nurturing and taking care of others. 3) sharing these nurturing or caring narratives, the participants lodged claims as valued persons who are living meaningful lives. | #Precarity #Surviving Violence and Exclusion #Existing with Risk  #Fracturing Identity |
| Gultekin 2014 | #1 family histories of violence, poverty, social isolation, #2 lack of informal support as contributing to homelessness 3# different perspectives between women and caseworkers --- *caseworker quotes not extracted* | #Precarity #Surviving Violence and Exclusion #Existing with Risk #Fracturing Identity |
| Kirkman2015 | 1) Pathways into homelessness, 2) Homelessness and mental health, 3) Women want stability, security, and safety, 4) Effects of shame and the perceived public discourse of homeless women, 5) Self-help and citizenship. | #Precarity #Surviving Violence and Exclusion #Existing with Risk |
| Lewinson2014 | #1Becoming homeless to escape violence and keep children safe(r)  #2 trauma, particularly physical and sexual abuse  #3 once homeless, increased risks of assault and exposure to violence #3 lack of agency | #Precarity #Surviving Violence and Exclusion #Existing with Risk # Parenting whilst homeless # Fracturing Identity |
| Mayock2015 | #1 Pervasiveness of patterns of repeat homelessness  #2 women’s relationship with homelessness and housing over time patterned in complex ways, and the spaces they occupied were often ambiguous and precarious.  #3 the impact of the absence of their children,  #4 violence as an ongoing feature of many of their lives.  #5 intimate partners emerge as strong drivers of their ongoing homelessness,  6# mobility patterns were deeply structured by institutional settings 7# accounts of infantilisation and in their perceptions of being treated like children | #Precarity #Surviving Violence and Exclusion #Existing with Risk # Fracturing Identity  # Parenting whilst homeless |
| McGrath 2023 | #1 Habitus of instability: anticipating crisis #2 Sofa surfing: utilising capital to navigate hidden homelessness #3 The field of intimate relationships 4# Domestic abuse: “ties that bind” 5# Services: a source of bonding or bridging capital? 6# social capital is a “double edged phenomenon” which impacts on health and housing vulnerability 7# the extent to which interpersonal relationships can play a role in shaming, stigma and exploitation for women 8#experiences of hostels perpetuated the homogeneity of their networks, often serving to deepen inequalities rather than ameliorate them | #Precarity #Surviving Violence and Exclusion #Existing with Risk # Fracturing Identity |
| Menih 2020 | #1 Narrative of risk for women experiencing homelessness; #2 women navigating risk on the streets by employing transiency, invisibility and squatting | #Precarity #Surviving Violence and Exclusion #Existing with Risk # Fracturing Identity |
| Moore2014 | 1) instability and stress, 2) a lack of control, 3) a homeless identity, 4) coping mechanisms, 5) adjustment and change. | #Precarity #Surviving Violence and Exclusion #Existing with Risk |
| Phipps2021A | five stages of resilience transition emerged: 1) The trauma of homelessness, 2) Finding hope and surviving, 3) Finding help, 4) Finding connection 4) Taking control. | #Precarity #Surviving Violence and Exclusion #Existing with Risk |
| Phipps 2021B | Recovery from homelessness is the overarching theme being presented, described using five subthemes: Finding the right house, Making a house a home, Connection, Building confidence and Helping others | #Precarity #Surviving Violence and Exclusion #Existing with Risk |
| Price and Glorney 2022 | Two super-ordinate themes emerged: 1) victimisation and trauma 2) the group and the individual. | #Precarity #Surviving Violence and Exclusion #Existing with Risk # Fracturing Identity |
| Salem and MaPham 2015 | (1) health care needs and challenges experienced)); (2) perspectives on sexual decision making; (3) employment difficulties; (4) existing support systems; and (5) development of future program plan | #Precarity #Surviving Violence and Exclusion #Existing with Risk |
| Salem 2013 | 1) traumatic life experiences 2) lack of access to health care 3) lack of self care knowledge 4) staying in touch for ongoing support | #Precarity #Surviving Violence and Exclusion #Existing with Risk |
| Salsi 2017 | occupational 'lives’ transitional journeys comprising five subthemes: 1) seeking safety and stability, 2) being sheltered, 3) shaping one’s identity, 4) developing resilience, and 50 engaging in contemplation, contribution, and connectedness through occupation | #Precarity #Surviving Violence and Exclusion #Existing with Risk |
| Schmid t2015 | 1) trajectory of women’s mental health and homelessness, - Four overarching themes emerged from women’s descriptions of the trajectory of their homelessness and their experience of accessing services: (a) unresolved trauma, (b) poverty and social exclusion, (c) inability to find and maintain housing and (d) ineffective services vicious cycles | #Precarity #Surviving Violence and Exclusion #Existing with Risk |
| Sutherland 2022 | safe accommodation; financial insecurity; experience of trauma and abuse; stigma, embarrassment and fear of being judged; the health impact of not fulfilling their role as family nurturer; mental health; complex interaction of physical and mental health issues; healthcare costs; and the need for ongoing psychosocial and healthcare support once housed | #Precarity #Surviving Violence and Exclusion #Existing with Risk |
| Tutty 2014 | violence as pathway into homelessness, violence as mechanism of control -three main routes into homelessness: the abuse from their partners, alcohol and drug abuse, and being discharged from an institution. typical scenario, according to several interviewees, consisted of living in a continuous state of uncertainty, fear of being left out on the street under unsafe circumstances. | #Precarity #Surviving Violence and Exclusion #Existing with Risk |
| Van Berkum 2019 | 1) On the Margins; 2) Feeling at Home; 3) Mighty Women; 4) Safety; Creating Home | #Precarity #Surviving Violence and Exclusion #Existing with Risk |
| Warburton2018 | Relational factors that contributed to becoming homeless. | #Precarity #Surviving Violence and Exclusion #Existing with Risk |
| Wilson 2015 | Four major themes emerged: (a) unstable/insecure housing over time, (b) limited support, (c) survival, and (d) mental health. | #Precarity #Surviving Violence and Exclusion #Existing with Risk |
