# Supplementary material for: A meta-ethnographic systematic review of women’s experiences of homelessness in high income environments
Source: PLoS One. 2026 Jan 20;21(1):e0339371. doi: 10.1371/journal.pone.0339371 (PMC12818621; doi:10.1371/journal.pone.0339371)
Supplement: S6 Appendix — (DOCX) [file pone.0339371.s006.docx]

| No | Authors | Title | Published Year | Notes |
| --- | --- | --- | --- | --- |
| 1 | Szeintuch, S; Spector-Mersel, G; Maor, T; Metzer, R | "I Was Born Into a Nightmare": The Chaotic Life Trajectories of Young Women Experiencing Homelessness | 2023 | Exclusion reason: Wrong outcomes |
| 2 | McCormack, F; Fedorowicz, S | Women, homelessness and multiple disadvantage in Stoke-on-Trent: The need for safe places in the context of wider health and social inequalities | 2022 | Exclusion reason: Wrong outcomes; |
| 3 | Benbow, Sarah | Mothers experiencing homelessness: Social exclusion, resistance, and health | 2023 | Exclusion reason: not peer reviewed publication- dissertation; |
| 4 | Thomas, Natalie; Menih, Helena | Negotiating multiple stigmas: Substance use in the lives of women experiencing homelessness | 2022 | Exclusion reason: Wrong outcomes; |
| 5 | Rudolph, Kaila A; Stewart, Madeline; Christina P.C. Borba | "Shelter is Stressing Me Out": Challenges Meeting Health Care Needs of Older Adults in Congregate Shelters | 2023 | Exclusion reason: Wrong patient population; |
| 6 | Hess, Carolin | Choosing invisibility? Exploring service (dis)engagement of women experiencing multiple disadvantage | 2023 | Exclusion reason: Wrong patient population; |
| 7 | Theobald, Jacqui; Watson, Juliet; Haylett, Freda; Murray, Suellen | Supporting Pregnant Women Experiencing Homelessness | 2023 | Exclusion reason: Wrong outcomes; |
| 8 | Lenta, Malena; Jorgelina Di Iorio; JosÃ© Juan VÃ¡zquez | Stressful Life Events among Women Living Homeless in Argentina | 2023 | Exclusion reason: Wrong setting; |
| 9 | Dickins, Kirsten A; Reed, Monique; Paun, Olimpia; Swanson, Barbara; Karnik, Niranjan S | Biopsychosocial Model of Traumatic Stress Symptoms in Women Experiencing Homelessness: A Qualitative Descriptive Study | 2023 | Exclusion reason: Unclear who produced which data; |
| 10 | Benbow, Sarah | Mothers experiencing homelessness: Social exclusion, resistance, and health | 2023 | Exclusion reason: Not a peer reviewed publication; |
| 11 | Vazquez, J. J.; Cabrera, A.; Panadero, S. | Evolution of the Accommodation Situation Among Women Living Homeless in Madrid, Spain: A Longitudinal Study | 2022 | Exclusion reason: Does not include qualitative data from women experiencing homelessness; |
| 12 | Vazquez, J. J.; Cabrera, A.; Panadero, S. | Access to employment and the labor market among women living homeless in Madrid, Spain | 2022 | Exclusion reason: focus of data presented is NOT on experiences of/relating to homelessness; |
| 13 | Trout, K. K.; Ayyagari, S.; Grube, W. C. | In Our Own Voices: The Lived Experience of Sex Workers in Philadelphia who Identify as Women | 2022 | Exclusion reason: focus of data presented is NOT on experiences of/relating to homelessness; |
| 14 | Toolis, Erin; Dutt, Anjali; Wren, Alexander; Jacksonâ€Gordon, Rachel | It's a place to feel like part of the community': Counterspace, inclusion, and empowerment in a dropâ€in center for homeless and marginalized women | 2022 | Exclusion reason: Wrong outcomes; |
| 15 | Thomas, N.; Menih, H. | Negotiating Multiple Stigmas: Substance Use in the Lives of Women Experiencing Homelessness | 2022 | Exclusion reason: Wrong study design; |
| 16 | Phipps, M.; Dalton, L.; Maxwell, H.; Cleary, M. | More than a house: Women's recovery from homelessness in Australia | 2022 | Exclusion reason: Wrong outcomes; |
| 17 | Mantler, T.; Jackson, K. T.; Walsh, E. J.; Tobah, S.; Shillington, K.; Jackson, B.; Soares, E. | Sharing Personal Experiences of Accessibility and Knowledge of Violence: A Qualitative Study | 2022 | Exclusion reason: Wrong outcomes; |
| 18 | Kitson, C.; Haines, M.; O'Byrne, P. | Understanding the Perspectives of Women Who Use Intravenous Drugs and are Experiencing Homelessness in an Urban Centre in Canada: An Analysis of Ethnographic Data | 2022 | Exclusion reason: Wrong outcomes; |
| 19 | Gonzalez, K. A.; Abreu, R. L.; Rosario, C. C.; Koech, J. M.; Lockett, G. M.; Lindley, L. | "A center for trans women where they help you": Resource needs of the immigrant Latinx transgender community | 2022 | Exclusion reason: Wrong outcomes; |
| 20 | Deal, E.; Hawkins, M.; Del Carmen Graf, M.; Dressel, A.; Ruiz, A.; Pittman, B.; Schmitt, M.; Krueger, E.; Lopez, A. A.; Mkandawire-Valhmu, L.; Kako, P. | Centering Our Voices: Experiences of Violence Among Homeless African American Women | 2022 | Exclusion reason: Wrong outcomes; |
| 21 | Berg, M. | Women Refugees' Media Usage: Overcoming Information Precarity in Germany | 2022 | Exclusion reason: Wrong patient population; |
| 22 | Zehetmair, C.; Kindermann, D.; Tegeler, I.; Derreza-Greeven, C.; Cranz, A.; Friederich, H. C.; Nikendei, C. | A Qualitative Evaluation of a Mother and Child Center Providing Psychosocial Support to Newly Arrived Female Refugees in a Registration and Reception Center in Germany | 2021 | Exclusion reason: Wrong outcomes; |
| 23 | Schultz-Krohn, Winifred; Winter, Emily; Mena, Carina; Roozeboom, Alison; Vu, Lisa | The Lived Experience of Mothers Who Are Homeless and Participated in an Occupational Therapy Leisure Craft Group | 2021 | Exclusion reason: focus of data presented is NOT on experiences of/relating to homelessness; |
| 24 | Ryan-DeDominicis, T. | A Case Study Using Shame Resilience Theory: Walking Each Other Home | 2021 | Exclusion reason: Does not include qualitative data from women experiencing homelessness; |
| 25 | Rivas-Rivero, E.; Panadero, S.; Vazquez, J. J. | Intimate partner sexual violence and violent victimisation among women living homeless in Madrid (Spain) | 2021 | Exclusion reason: Does not include qualitative data from women experiencing homelessness; |
| 26 | Reid, N.; Kron, A.; Rajakulendran, T.; Kahan, D.; Noble, A.; Stergiopoulos, V. | Promoting Wellness and Recovery of Young Women Experiencing Gender-Based Violence and Homelessness: The Role of Trauma-Informed Health Promotion Interventions | 2021 | Exclusion reason: Wrong outcomes; |
| 27 | Racette, Elise H.; Fowler, Christopher A.; Faith, Laura A.; Geis, Bill D.; Rempfer, Melisa V. | Characteristics of trauma among women experiencing homelessness: An exploratory cluster analysis | 2021 | Exclusion reason: Wrong study design; |
| 28 | Mostowska, M.; Debska, K. | The Conspicuous Hidden Curriculum and Young Women's Daily Lives in Polish Crisis Accommodation | 2021 | Exclusion reason: focus of data presented is NOT on experiences of/relating to homelessness; |
| 29 | Kitson, C.; O'Byrne, P. | The Experience of Violence Against Women Who Use Injection Drugs: An Exploratory Qualitative Study | 2021 | Exclusion reason: Wrong outcomes; |
| 30 | Gruer, C.; Hopper, K.; Smith, R. C.; Kelly, E.; Maroko, A.; Sommer, M. | Seeking menstrual products: a qualitative exploration of the unmet menstrual needs of individuals experiencing homelessness in New York City | 2021 | Exclusion reason: focus of data presented is NOT on experiences of/relating to homelessness; |
| 31 | Gregory, Katie; Nnawulezi, Nkiru; Sullivan, Cris M. | Understanding how domestic violence shelter rules may influence survivor empowerment | 2021 | Exclusion reason: Wrong outcomes; |
| 32 | Granfelt, R.; Turunen, S. | Women on the Border between Home and Homelessness: Analysing Worker-Client Relationship | 2021 | Exclusion reason: focus of data presented is NOT on experiences of/relating to homelessness; |
| 33 | Cordero-Ramos, N.; Munoz-Bellerin, M. | Human Rights in front of the Mirror. Narratives of Homeless Women in the Exercise of Their Capacities | 2021 | Exclusion reason: main article not in English language; |
| 34 | Beasley, Monica | Qualitative study on improving the effectiveness of homeless services in Vermilion County, Illinois | 2021 | Exclusion reason: Not a peer reviewed publication; |
| 35 | Yu, B.; Montgomery, A. E.; True, G.; Cusack, M.; Sorrentino, A.; Chhabra, M.; Dichter, M. E. | The Intersection of Interpersonal Violence and Housing Instability: Perspectives From Women Veterans | 2020 | Exclusion reason: focus of data presented is NOT on experiences of/relating to homelessness; |
| 36 | Vazquez, J. J.; Panadero, S.; Garcia-Perez, C. | Immigrant Women Living Homeless in Madrid (Spain) | 2020 | Exclusion reason: Wrong study design; |
| 37 | Vazquez, J. J.; Panadero, S. | Meta-stereotypes among women living homeless: Content, uniformity, and differences based on gender in Madrid, Spain | 2020 | Exclusion reason: Wrong study design; |
| 38 | Richards, J.; Oudshoorn, A.; Misener, L. | Social Inclusion Through Sport for Women Experiencing Homelessness | 2020 | Exclusion reason: focus of data presented is NOT on experiences of/relating to homelessness; |
| 39 | Reppond, Harmony A.; Bullock, Heather E. | Reclaiming 'good motherhood': US mothersâ€™ critical resistance in family homeless shelters | 2020 | Exclusion reason: Wrong outcomes; |
| 40 | Pardo, A. A.; Ramirez, J. P.; Martinez, A. I. | HOMELESS WOMEN IN SPAIN. NARRATIVES ABOUT GENDER, SOCIAL VULNERABILITY AND WELFARE SCHEME'S EFFECTS | 2020 | Exclusion reason: main article not in English language; |
| 41 | Mostowska, M.; Debska, K. | An ambiguous hierarchy of inequalities. The political intersectionality of older women's homelessness in Poland | 2020 | Exclusion reason: Does not include qualitative data from women experiencing homelessness; |
| 42 | Monari, E.; Booth, R.; Harerimana, B.; Forchuk, C. | The Experiences of Migration among Homeless Male and Female Psychiatric Survivors | 2020 | Exclusion reason: Cannot report women's accounts ; |
| 43 | Milaney, K.; Williams, N.; Lockerbie, S. L.; Dutton, D. J.; Hyshka, E. | Recognizing and responding to women experiencing homelessness with gendered and trauma-informed care | 2020 | Exclusion reason: Does not include qualitative data from women experiencing homelessness; |
| 44 | Milaney, K.; Tremblay, R.; Bristowe, S.; Ramage, K. | Welcome to Canada: Why Are Family Emergency Shelters 'Home' for Recent Newcomers? | 2020 | Exclusion reason: Wrong outcomes; |
| 45 | McCarthy, L. | Homeless women, material objects and home (un)making | 2020 | Exclusion reason: focus of data presented is NOT on experiences of/relating to homelessness; |
| 46 | Macdonald, M.; Kane, D.; Williams, J. | Protecting women with multiple and complex needs from gendered violence: impediments to obtaining and maintaining safe and secure accommodation in a European context | 2020 | Exclusion reason: Wrong outcomes; |
| 47 | Kenyon, Katherine M.; Hiebert-Murphy, Diane; Ristock, Janice; Medved, Maria I. | The process of empowerment reflected in women's narratives of their stay in a domestic violence shelter | 2020 | Exclusion reason: focus of data presented is NOT on experiences of/relating to homelessness; (2023-04-04 05:30:56) : asked via inter library loan ; Anne Cronin (2023-03-28 06:16:29) : don't have access to text; (2023-03-07 03:51:14) : requested full text from author; |
| 48 | Felder, S.; Delany, P. J. | The life course of homeless female Veterans: Qualitative study findings | 2020 | Exclusion reason: Wrong outcomes; |
| 49 | Fauci, Jennifer E.; Goodman, Lisa A. | "You Don't Need Nobody Else Knocking you Down": Survivor-Mothers' Experiences of Surveillance in Domestic Violence Shelters | 2020 | Exclusion reason: Wrong outcomes; |
| 50 | Broll, R.; Huey, L. | "Every Time I Try to Get Out, I Get Pushed Back": The Role of Violent Victimization in Women's Experience of Multiple Episodes of Homelessness | 2020 | Exclusion reason: Does not include qualitative data from women experiencing homelessness; |
| 51 | Babatunde-Sowole, O. O.; Power, T.; Davidson, P. M.; DiGiacomo, M.; Jackson, D. | Health screening and preventative health care in refugee women: A qualitative analysis | 2020 | Exclusion reason: focus of data presented is NOT on experiences of/relating to homelessness; |
| 52 | Vazquez, J. J.; Panadero, S. | Suicidal Attempts and Stressful Life Events Among Women in a Homeless Situation in Madrid (Spain) | 2019 | Exclusion reason: Does not include qualitative data from women experiencing homelessness; |
| 53 | Shah, P.; Koch, T.; Singh, S. | The attitudes of homeless women in London towards contraception | 2019 | Exclusion reason: Wrong outcomes; |
| 54 | Mueller, Mary Ann | Experiences and attitudes of older homeless women toward healthcare access | 2019 | Exclusion reason: Not a peer reviewed publication; |
| 55 | Kenny, D. J.; Yoder, L. H. | A picture of the older homeless female veteran: A qualitative, case study analysis | 2019 | Exclusion reason: Wrong outcomes; |
| 56 | Hanley, J.; Ives, N.; Lenet, J.; Hordyk, S. R.; Walsh, C.; Ben Soltane, S.; Este, D. | Migrant women's health and housing insecurity: an intersectional analysis | 2019 | Exclusion reason: Wrong outcomes; |
| 57 | Groton, Danielle B.; Radey, Melissa | Social networks of unaccompanied women experiencing homelessness | 2019 | Exclusion reason: Wrong outcomes; |
| 58 | Gordon, A. C.; Lehane, D.; Burr, J.; Mitchell, C. | Influence of past trauma and health interactions on homeless women's views of perinatal care: a qualitative study | 2019 | Exclusion reason: focus of data presented is NOT on experiences of/relating to homelessness; |
| 59 | Glumbikova, K.; Gojova, A.; Grundelova, B. | Critical reflection of the reintegration process through the lens of gender oppression: the case of social work with mothers in shelters | 2019 | Exclusion reason: Wrong outcomes; |
| 60 | Fisher, Elisa M.; Stylianou, Amanda M. | To stay or to leave: Factors influencing victimsâ€™ decisions to stay or leave a domestic violence emergency shelter | 2019 | Exclusion reason: Wrong outcomes; |
| 61 | Dawes, J.; Sanders, C.; Allen, R. | "A Mile in Her Shoes": A qualitative exploration of the perceived benefits of volunteer led running groups for homeless women | 2019 | Exclusion reason: Wrong outcomes; |
| 62 | Buchbinder, Eli; Karayanni, Nisreen Georgre | The Aftermath of Shelter: Abused Arab Women in Israel | 2019 | Exclusion reason: Wrong outcomes; |
| 63 | Vaughn, Rachel Elizabeth | The complex cycle of interpersonal violence and homelessness: Perspectives of homeless women | 2018 | Exclusion reason: unpublished/not peer reviewed work; |
| 64 | McCarthy, Lindsey Jayne | (Re) negotiating the self : Homeless women's constructions of home, homelessness and identity | 2018 | Exclusion reason: Not a peer reviewed publication; |
| 65 | McCarthy, Lindsey | (Re)conceptualising the boundaries between home and homelessness: the unheimlich | 2018 | Exclusion reason: Wrong outcomes; |
| 66 | Hutt, E.; Albright, K.; Dischinger, H.; Weber, M.; Jones, J.; O'Toole, T. P. | Addressing the Challenges of Palliative Care for Homeless Veterans | 2018 | Exclusion reason: Cannot report women's accounts ; |
| 67 | Flynn, Catherine; Damant, Dominique; Lapierre, Simon; Lessard, GeneviÃ¨ve; Gagnon, Charlotte; Couturier, Vanessa; Couturier, PÃ©nÃ©lope | When structural violences create a context that facilitates sexual assault and intimate partner violence against street-involved young women | 2018 | Exclusion reason: Wrong outcomes; |
| 68 | Darab, S.; Hartman, Y.; Holdsworth, L. | What women want: single older women and their housing preferences | 2018 | Exclusion reason: focus of data presented is NOT on experiences of/relating to homelessness; |
| 69 | Tutty, Leslie M.; Augusta-Scott, Tod; Scott, Katreena; Tutty, Leslie M. | A place to go to when I had no place to go to': Journeys of violence against women's emergency shelter residents | 2017 | Exclusion reason: Wrong outcomes; |
| 70 | Sharam, A. | The Voices of Midlife Women Facing Housing Insecurity in Victoria, Australia | 2017 | Exclusion reason: Wrong patient population; |
| 71 | Mantler, T.; Wolfe, B. | A rural shelter in Ontario adapting to address the changing needs of women who have experienced intimate partner violence: a qualitative case study | 2017 | Exclusion reason: focus of data presented is NOT on experiences of/relating to homelessness; |
| 72 | Kelly, L.; Luxford, Y. | Come walk with me: Homelessness, nursing and engaged care | 2017 | Exclusion reason: Does not include qualitative data from women experiencing homelessness; |
| 73 | JuandÃ³-Prats, Clara | Health Care Access and Utilization by Young Mothers Experiencing Homelessness: A Bourdieusian Analysis with an Arts-Based Approach | 2017 | Exclusion reason: Not a peer reviewed publication; |
| 74 | Gultekin, L.; Brush, B. L. | In Their Own Words: Exploring Family Pathways to Housing Instability | 2017 | Exclusion reason: Wrong outcomes; |
| 75 | Baker, Kathleen R. | Young women's perceived challenges: Living independently after aging out of foster care | 2017 | Exclusion reason: Not a peer reviewed publication; |
| 76 | Auffrey, Monique; Tutty, Leslie M.; Wright, Alysia C.; Augusta-Scott, Tod; Scott, Katreena; Tutty, Leslie M. | Preventing homelessness for women who leave abusive partners: A shelter-based 'housing first' program | 2017 | Exclusion reason: Cannot report women's accounts ; |
| 77 | Abbott, P.; Magin, P.; Davison, J.; Hu, W. | Medical homelessness and candidacy: women transiting between prison and community health care | 2017 | Exclusion reason: focus of data presented is NOT on experiences of/relating to homelessness; |
| 78 | Wetegrove-Romine, Amanda | Ask us what's best for us:' what counseling psychologists can learn from women experiencing homelessness | 2016 | Exclusion reason: Not a peer reviewed publication; |
| 79 | Weinrich, S.; Hardin, S.; Glaser, D.; Barger, M.; Bormann, J.; Lizarraga, C.; Terry, M.; Criscenzo, J.; Allard, C. B. | Assessing sexual trauma histories in homeless women | 2016 | Exclusion reason: Wrong study design; |
| 80 | Watson, J. | Gender-based violence and young homeless women: femininity, embodiment and vicarious physical capital | 2016 | Exclusion reason: Wrong outcomes; |
| 81 | Sichi, Mary | Mothers in resistance: An exploration of the perspectives of women with Child Protective Services experiences | 2016 | Exclusion reason: Not a peer reviewed publication; |
| 82 | Reppond, Harmony A. | When 'you can't get to where you're going:' Mothers' experiences in family homeless shelters | 2016 | Exclusion reason: Not a peer reviewed publication; |
| 83 | Otte, Kristen A. | Exploring themes of moral injury and resilience among women in a transitional living center | 2016 | Exclusion reason: Not a peer reviewed publication; |
| 84 | Mountain, Andree M. | The lived experience of battered women in transitional housing | 2016 | Exclusion reason: Not a peer reviewed publication; |
| 85 | Lyons, T.; Krusi, A.; Pierre, L.; Smith, A.; Small, W.; Shannon, K. | Experiences of Trans Women and Two-Spirit Persons Accessing Women-Specific Health and Housing Services in a Downtown Neighborhood of Vancouver, Canada | 2016 | Exclusion reason: Cannot report women's accounts ; |
| 86 | Kunkel, Adrianne; Guthrie, Jennifer A. | Survivor: Women's stories of navigation and tensions in a domestic violence shelter | 2016 | Exclusion reason: Wrong outcomes; |
| 87 | Grace, M.; Malone, J.; Murphy, A. | WAND: An Activity Program for Women in a Rooming House | 2016 | Exclusion reason: Wrong outcomes; |
| 88 | De Antoni, Clarissa; Assmann Ruas MunhÃ³s, Aline | The institutional violence and structural violence experienced by homeless women | 2016 | Exclusion reason: Wrong outcomes; |
| 89 | Torchalla, I.; Linden, I. A.; Strehlau, V.; Neilson, E. K.; Krausz, M. | "Like a lots happened with my whole childhood": violence, trauma, and addiction in pregnant and postpartum women from Vancouver's Downtown Eastside | 2015 | Exclusion reason: focus of data presented is NOT on experiences of/relating to homelessness; |
| 90 | Quinn, Katherine; Young, Staci; Thomas, Dave; Baldwin, Brennan; Paul, Melanie | The role of supportive housing for HIV-positive mothers and their children | 2015 | Exclusion reason: focus of data presented is NOT on experiences of/relating to homelessness; |
| 91 | Petersen, M. | Addressing older women's homelessness: service and housing models | 2015 | Exclusion reason: Cannot report women's accounts ; |
| 92 | Moxley, David P.; Washington, Olivia G. M.; Crystal, Jennifer | The relevance of four narrative themes for understanding vulnerability among homeless older African-American women | 2015 | Exclusion reason: Does not include qualitative data from women experiencing homelessness; |
| 93 | Long, Susan M. | Navigating homelessness and navigating abuse: How homeless mothers find transitional housing while managing intimate partner violence | 2015 | Exclusion reason: Wrong outcomes; |
| 94 | Ha, Y.; Narendorf, S. C.; Santa Maria, D.; Bezette-Flores, N. | Barriers and facilitators to shelter utilization among homeless young adults | 2015 | Exclusion reason: Wrong patient population; |
| 95 | Fortin, R.; Jackson, S. F.; Maher, J.; Moravac, C. | I WAS HERE: young mothers who have experienced homelessness use Photovoice and participatory qualitative analysis to demonstrate strengths and assets | 2015 | Exclusion reason: Wrong outcomes; |
| 96 | Fordham, M. | The lived experience of homeless women: insights gained as a specialist practitioner | 2015 | Exclusion reason: Wrong outcomes; |
| 97 | Erdem, Gizem | Can a house become more than a home? effects of housing assistance and supportive services on promoting capabilities among homeless mothers | 2015 | Exclusion reason: Cannot report women's accounts ; |
| 98 | Coleman, Michelle R. | The journey from homelessness to housing: The lived experiences of married african american women with children residing in supportive housing | 2015 | Exclusion reason: Not a peer reviewed publication; |
| 99 | Buchbinder, Eli; Karayanni, Nisreen George | Rejection and choice: Arab battered women coping with stigmatization after leaving battered womenâ€™s shelters in Israel | 2015 | Exclusion reason: Wrong outcomes; |
| 100 | Shechory-Bitton, Mally | A glimpse into the world of battered ultra-Orthodox Jewish women in Israel: A follow-up study on women who resided in a shelter | 2014 | Exclusion reason: Wrong outcomes; |
| 101 | Kennedy, S.; Grewal, M.; Roberts, E. M.; Steinauer, J.; Dehlendorf, C. | A qualitative study of pregnancy intention and the use of contraception among homeless women with children | 2014 | Exclusion reason: Wrong outcomes; |
| 102 | Jonker, Irene E.; Jansen, Carinda C. J. M.; Christians, Milou G. M.; Wolf, Judith R. L. M. | Appropriate care for shelter-based abused women: Concept mapping with Dutch clients and professionals | 2014 | Exclusion reason: Does not include qualitative data from women experiencing homelessness; |
| 103 | Huey, Laura; Hryniewicz, Danielle; Fthenos, Georgios | I had a lot of anger and thatâ€™s what kind of led me to cutting myselfâ€™: Employing a social stress framework to explain why some homeless women self-injure | 2014 | Exclusion reason: Wrong outcomes; |
| 104 | Anderson, Debra Gay; Fallin, Amanda; Al-Modallal, Hanan | Workplace violence experiences of homeless women and women residing in battered women shelters | 2014 | Exclusion reason: focus of data presented is NOT on experiences of/relating to homelessness; |
| 105 | Wendt, S.; Baker, J. | Aboriginal Women's Perceptions and Experiences of a Family Violence Transitional Accommodation Service | 2013 | Exclusion reason: Wrong outcomes; |
| 106 | Waldbrook, Natalie | Formerly homeless, older women's experiences with health, housing, and aging | 2013 | Exclusion reason: Wrong patient population; |
| 107 | Huey, L.; Fthenos, G.; Hryniewicz, D. | "If something happened, I will leave it, let it go and move on": resiliency and victimized homeless women's attitudes toward mental health counseling | 2013 | Exclusion reason: Wrong outcomes; |
| 108 | Holger-Ambrose, B.; Langmade, C.; Edinburgh, L. D.; Saewyc, E. | The illusions and juxtapositions of commercial sexual exploitation among youth: identifying effective street-outreach strategies | 2013 | Exclusion reason: focus of data presented is NOT on experiences of/relating to homelessness; |
| 109 | Clark, Angela; Lee, Rebecca C. | Transitioning through family homelessness and the effect of substance abuse on social support systems | 2013 | Exclusion reason: Wrong outcomes; |
| 110 | Cederbaum, J. A.; Wenzel, S. L.; Gilbert, M. L.; Chereji, E. | The HIV Risk Reduction Needs of Homeless Women in Los Angeles | 2013 | Exclusion reason: Wrong outcomes; |
| 111 | Brann, Suzette Emerald | Triple jeopardy---homeless, black, and female: Pathways to and from crime and homelessness | 2013 | Exclusion reason: Not a peer reviewed publication; |
| 112 | Biederman, Donna J. | Understanding the experience of interacting with service providers from the perspective of homeless women: A phenomenological study | 2013 | Exclusion reason: Not a peer reviewed publication; |
| 113 | Stringer, M.; Averbuch, T.; Brooks, P. M.; Jemmott, L. S. | Response to homeless childbearing women's health care learning needs | 2012 | Exclusion reason: focus of data presented is NOT on experiences of/relating to homelessness; |
| 114 | Shimmin, Jessica | Homemaking: Gender, safety, and place in Massachusetts battered women's shelters | 2012 | Exclusion reason: unpublished/not peer reviewed work; |
| 115 | Oliver, Vanessa; Cheff, Rebecca | Sexual Health: The Role of Sexual Health Services Among Homeless Young Women Living in Toronto, Canada | 2012 | Exclusion reason: Wrong outcomes; |
| 116 | Nettleton, S.; Neale, J.; Stevenson, C. | Sleeping at the margins: a qualitative study of homeless drug users who stay in emergency hostels and shelters | 2012 | Exclusion reason: Wrong patient population; |
| 117 | Mellor, R.; Lovell, A. | The lived experience of UK street-based sex workers and the health consequences: an exploratory study | 2012 | Exclusion reason: Wrong outcomes; |
| 118 | Lee, Rebecca C. | Family homelessness viewed through the lens of health and human rights | 2012 | Exclusion reason: Wrong outcomes; |
| 119 | Knapton, Cindy; Walsh, Christine A.; Browne-Miller, Angela | Violence in the lives of homeless women: Implications for shelter design | 2012 | Exclusion reason: pre 2012 (shouldnt have made to full text ); |
| 120 | Hamilton, Alison B.; Poza, Ines; Hines, Vivian; Washington, Donna L. | Barriers to psychosocial services among homeless women veterans | 2012 | Exclusion reason: Wrong outcomes; |
| 121 | Eaton, A. A., Stephens, D. P., Ruvalcaba, Y., Banks, J., & Sundari Foundation Inc. | A culture of care: How Lotus House Women's  Shelter heals program participants through  genuineness, space, high expectations, dignity,  individualized attention, and community | 2022 | Excluded at metaethnography meeting round one review by third reviewer as no agreement in first round |
| 122 | Graf, M. D., Dressel, A., Schmitt, M., Deal, E., Pittman, B., Lopez, A., Kako, P., & Mkandawire-Valhmu, L. | “I was broken in so many different ways”: The intersection of complex factors impacting homeless and at-risk African American women’s mental health and well-being. | 2022 | Excluded at metaethnography meeting round one review by third reviewer as no agreement in first round |
| 123 | Moxley, D. P., & Washington, O. G. M. | Souls in Extremis: Enacting Processes of Recovery from Homelessness Among Older African American Women | 2016 | Excluded at metaethnography meeting round one review by third reviewer as no agreement in first round |
| 124 | Dressel, A., Hawkins, M., Lopez, A. A., Pittman-McGee, B., Kako, P., Gakii, D., & Mkandawire-Valhmu, L. | Nia Imani Model of Care's Impact on Homeless African-American Women | 2020 | Exclusion reason: focus of data presented is NOT on experiences of/relating to homelessness; |
