# Supplementary material for: A meta-ethnographic systematic review of women’s experiences of homelessness in high income environments
Source: PLoS One. 2026 Jan 20;21(1):e0339371. doi: 10.1371/journal.pone.0339371 (PMC12818621; doi:10.1371/journal.pone.0339371)
Supplement: Attachment — Submitted filename: Reviewer Comment_Plos One.docx [file pone.0339371.s008.docx]

**A Meta-ethnographic systematic review of Women’s experiences of homelessness high-income environments**

**Reviewer Comments**

Thank you for this important research that aims to shed light on the invisibility of women’s homelessness in high-income countries. Because the majority of women deal with issues of abuse, shame, and stigma regarding their household dynamics, they are unlikely to be seen at public shelters or drop-ins, resulting in a lot of undocumented cases of women’s homelessness issues. The few that end up in shelters or seek support face structural challneges and may be excluded from research about homelessness. This meta-ethnography analysis is timely and critical in adding to the literature about women and homelessness in high-income countries. While the study has merit and contributes to knowledge, authors must address the following comments to get the manuscript to publication.

**Introduction**

The authors need to expand the introduction by providing a justification for their chosen context—high-income countries—and addressing why we need to understand the gendered homelessness situation in these countries. What is currently known about this issue in the study contexts, and why is it important?

Line 45 – please expand ‘multiple exclusion homelessness (MEH) and provide examples.

Lines 64-68 – why high-income countries; explain. While it is essential that the authors mention using the World Bank classification of high-income countries, they need to state the countries under review.

**Methods**

The authors claim to have used Noblit and Hare's (1998) meta-ethnography framework but fail to explain how they followed the seven steps Noblit and Hare provided. The authors need to expand the methods section to include what they did at every stage. If the authors did otherwise, they must justify and explain any modification.

Lines 108-111: The authors said they completed searches for 10 years (2012-2022) and then decided to expand the search to include literature from 2022 through January 2024 but did not explain why this was done. First, why did you decide on 10 years? Second, what led to the inclusion of additional data? These things need to be clearly explained.

Lines 119-120 – were there specific things the author excluded and/or included, and did any decision about the inclusion/exclusion criteria affect the data analysis?

**Results**

The authors decided to capture some information about the methods under the results section, which affected the flow of the paper. I suggest moving information from lines 122-158 to the methods section and making a link to Noblit and Hare’s framework.

Line 126 – please explain this statement: ‘34 papers reporting on 32 studies in the review.’ Does this statement suggest two papers written in the same context? If so, clearly state that. The reverse is captured in Figure 1.

Line 159 – is where the result section should begin.

Lines 124-126—Please review the numbers and compare them to the ones in the figure. There are discrepancies. For example, the authors said they screened 3332, but in the diagram, 3594 is captured. Also, they said the final list under review is 159, but in the figure, 158 is captured. Please make the necessary corrections. But if this is not an error, the authors should explain this discrepancy.

Line 171 – ‘five were mixed methods’ – but Figure 1 records 2. Which one is correct?

Line 169 – list the 9 high-income countries.

Lines 202-203 – the title of the figure/image should come under the image.

Lines 205-207 – there should be a signal to the theoretical framework in the Introduction section. This should tie in with the lit review on the motivation for the study; please revise accordingly.

Line 223 – there is something missing in the sentence…’they survive…’

Lines 247-249 – there should be a note about the use of quotes either in the methods section or the first paragraph in the results section.

**Discussion**

How has the social ecological model helped explain the results; that is missing in the discussion section.
