# Supplementary material for: A meta-ethnographic systematic review of women’s experiences of homelessness in high income environments
Source: PLoS One. 2026 Jan 20;21(1):e0339371. doi: 10.1371/journal.pone.0339371 (PMC12818621; doi:10.1371/journal.pone.0339371)
Supplement: Attachment — Submitted filename: Response to ReviewersMay25 (2).docx [file pone.0339371.s009.docx]

Editorial requests from 30.3.25

| Editor and Reviewer Comments | Author Comments |
| --- | --- |
| 1. Your abstract cannot contain citations. Please remove any existing citations from the abstract section. You may only include citations in the body text of the manuscript, and please ensure that they remain in ascending numerical order on first mention. | Thank you for your suggestions and we have revised the text as noted and updated in this section. |
| 2. Please ensure that you refer to Figure 2 in your text as, if accepted, production will need this reference to link the reader to the figure. | Thank you for your suggestions and we have revised the text as noted and updated in this section. |
| As required by our policy on Data Availability, please ensure your manuscript or supplementary information includes the following:  1. A numbered table of all 3322 studies identified in the literature search, including those that were excluded from the analyses.  a. For every excluded study, the table should list the reason(s) for exclusion.  b. If any of the included studies are unpublished, include a link (URL) to the primary source or detailed information about how the content can be accessed.  This information can be included in the main text, supplementary information, or relevant data repository. Please note that providing these underlying data is a requirement for publication in this journal, and if these data are not provided your manuscript might be rejected. | Many thanks for your suggested data availability comment. We understand the requirement for all empirical data reporting. However, this is highly unusual request and is not required for Cochrane systematic reviews per their MECIR documents and PRISMA reporting. The normal requirements for any review Cochrane or not, as per PRISMA guidelines is to provide a PRISMA table with the reasons for excluding full text papers. We have completed this information.  We have provided a supplemental file with the list of 124 citations and the linked reason for exclusion as captured in the Covidence platform. This is what is captured in this search platform . We would request Editorial advice to understand why an impossible ask is placed on ourselves as we would have to revisit the entire 3322 papers that we undertook title and abstract review only of to provide this.. We wish to publish in PLOS One and are grateful for considered response. |
