# Supplementary material for: A meta-ethnographic systematic review of women’s experiences of homelessness in high income environments
Source: PLoS One. 2026 Jan 20;21(1):e0339371. doi: 10.1371/journal.pone.0339371 (PMC12818621; doi:10.1371/journal.pone.0339371)
Supplement: Attachment — Submitted filename: Responsetoreviewers030925.docx [file pone.0339371.s010.docx]

School of Nursing Midwifery and Health Systems

University College Dublin

Ireland

3^rd^ September 2025

Dear Editor,
Many thanks for the welcomed decision and the positive responses from all three peer reviewers. We appreciate the opportunity to strengthen the methodological reporting and enhancing the limitations to our review. Please see the responses to the points raised by the reviewers as follows

1) All included studies come from urban or predominantly urban contexts; rural homelessness experiences are absent. Please, explicitly acknowledge this limitation in the Discussion, outlining implications for transferability of findings.
   **Response. Thank you we have added this on page 13.**
2) Almost half the included studies do not define “homelessness,” potentially undermining synthesis consistency.
   Hence, provide a critical reflection on how definitional variability may affect comparability, perhaps including a sensitivity analysis excluding studies without definitions. **Thank you and we have added a critical reflection on this matter on page 14.**
3) There is a large variation in data collection methods (single interview, ethnography, photovoice) and absence of reflexivity in most included studies. Please, discuss explicitly how methodological heterogeneity was handled in the synthesis to mitigate bias. **Thank you we have added this to the discussion on page 7.**
4) The time restriction (10 years) is described as “pragmatic” but lacks a strong methodological justification beyond contemporaneity. Please, provide stronger rationale or explore inclusion of older, high-quality studies in a sensitivity check. **Response. Thank you for this perspective. Our review over a 10 and now longer during peer review is a substantial body of evidence of women’s experiences. We did not aim to report studies from inception and similarly to other meta synthesis reported in PLOS One journal a limitation on years of search exists as we have clearly reported in the limitations section. We reject the view that another search is required.  We have added additional text that briefly explains that our initial search yielded over 5768 records and the rational for the decision at that point to restrict searches to 2012 onwards.  Our current search includes 12 years of data. This review was unfunded and is part of a doctoral research study. We identified examples of similar systemic reviews in PLOS One specifically that have restricted years and present a few both demonstrating meta-analysis and meta synthesis.**

**2024: limited search and no limitations section reported.** [**https://journals.plos.org/plosntds/article?id=10.1371/journal.pntd.0012718**](https://journals.plos.org/plosntds/article?id=10.1371/journal.pntd.0012718)

**2025: search years limited to 5 years.** [**https://journals.plos.org/globalpublichealth/article?id=10.1371/journal.pgph.0004483**](https://journals.plos.org/globalpublichealth/article?id=10.1371/journal.pgph.0004483)

**2025:  10 years search** [**https://journals.plos.org/globalpublichealth/article?id=10.1371/journal.pgph.0004272**](https://journals.plos.org/globalpublichealth/article?id=10.1371/journal.pgph.0004272)

**2025: 15 year search 2009 to 2024** [**https://journals.plos.org/plosone/article?id=10.1371/journal.pone.0322796**](https://journals.plos.org/plosone/article?id=10.1371/journal.pone.0322796)

**2025: 10 years and no limitations section.**

[**https://journals.plos.org/plosone/article?id=10.1371/journal.pone.0325683**](https://journals.plos.org/plosone/article?id=10.1371/journal.pone.0325683)

5) You state there were no significantly refutational studies, but this is unexpected in qualitative synthesis. So, revisit dataset to ensure potentially divergent findings are not being subsumed into dominant narratives; even subtle contradictions can enrich the synthesis.
   Intersectionality is acknowledged but underdeveloped; ethnicity, migration status, and disability receive little explicit attention despite likely importance.
   **Response. Thank you we have revisited our analysis and our interpretation is correct.  We have highlighted the paucity of detailed qualitative reporting from the collected studies including women who experience homelessness. We are transparent in our reporting and we have added to the discussion regarding intersectionality on page 9 and 20.**
6) Please, incorporate more targeted analysis of how overlapping identities shape the homelessness experience. **Response. Thank you and we have further added further commentary on Page 9 .**
7) Certain passages are emotionally powerful but verge on advocacy rather than analysis. Retain authenticity of participant voice but balance with analytical neutrality to align with journal expectations. **Response. Thank you for highlighting and we have revised the tone of some passages to address this.**

Best wishes

Maxine Radcliffe

RN NP MPH DTN Stage 2 Scholar UCD
